# Epoxy Nanocomposites with Carbon Nanotubes Produced by Floating Catalyst CVD

**DOI:** 10.3390/nano11051213

**Published:** 2021-05-04

**Authors:** Vladimir Z. Mordkovich, Stanislav V. Kondrashov, Aida R. Karaeva, Sergey A. Urvanov, Nikita V. Kazennov, Eduard B. Mitberg, Ekaterina A. Pushina

**Affiliations:** 1 Technological Institute for Superhard and Novel Carbon Materials, 7A Tsentralnaya Street, Troitsk, 108840 Moscow, Russia; karaevaar@tisnum.ru (A.R.K.); urvanov@tisnum.ru (S.A.U.); kazennov@infratechnology.ru (N.V.K.); mitbergeb@tisnum.ru (E.B.M.); katyazhu@tisnum.ru (E.A.P.); 2 Department of Chemistry, Moscow State University, 1-Str.3 Leninskie Gory, 119991 Moscow, Russia; 3 All-Russian Scientific Research Institute of Aviation Materials, 17 Radio Str., 105005 Moscow, Russia; stasru_59@mail.ru

**Keywords:** carbon, nanotube, epoxy, nanocomposite, curing, composite material

## Abstract

Epoxy nanocomposites with float catalysis-produced CNT felt as a filler were prepared. Parameters such as the curing process, glass transition of epoxynanocomposites, structure and morphology of CNT felt, initial epoxy composition, and epoxy nanocomposites were investigated. The influence of CNT felt on curing process in epoxy nanocomposites with different amounts of curing agent was determined. An exothermic reaction between the curing agent and the surface of CNTs was established. It was found that the structure of epoxy nanocomposites has a high degree of heterogeneity: the presence of fiber-like structures and individualized CNTs is observed together with the regions that are typical for CNTs that are fabricated via a catalytic chemical vapor deposition (CVD). Based on the studies performed, it is possible to predict the production of epoxy nanocomposites with outstanding mechanical and thermophysical properties. In particular, the uncured compositions already obtained in this work can be used for the manufacture of electrically conductive glass and carbon fiber reinforced plastics and functional coatings.

## 1. Introduction

Carbon nanotubes (CNTs) in the form of a nonwoven fabric (felt) can be used as nanofillers of structural polymer composite materials (PCM) and to achieve a high degree of filling in PCM [1]. One of the scalable methods for the synthesis of such CNTs is a method of float catalysis (FC-CVD), which implies introduction of a gasified catalyst precursor into a feed gas for CNT synthesis, which leads to formation of CNT aerogel in the end of the reaction [2]. The feed gas is injected into a CVD reactor at a temperature of about 1100–1200 °C. CNT aerogel is removed from the reactor by the carrier gas and can be reeled up onto a spool after the reaction. Several studies have shown a significant improvement in the properties of nanocomposites with CNTs obtained by the FC-CVD method after functionalization [3,4,5,6]. It is known that the presence of functional groups on the surface of CNTs leads to a change in the rate, thermal effect, and even the mechanism of the curing reaction [7]. Probably, the catalyst residues obtained during the synthesis at the surface of CNTs can also lead to a similar effect [8]. The literature and our own experience suggest that the introduction of nanotubes usually leads to an acceleration of the curing process, which produces certain difficulties in production of composites. Another important observation is that CNT introduction leads to rapid loss of fluidity of uncured composition with the rising content of CNT. The CNTs reported recently as a product of our own version of FC-CVD [9] are characterized by outstanding nanotube length (superlong nanotubes) and domination of double-walled nanotubes. The observations of these nanotubes suggested that their interaction with epoxy compositions is different in many ways. The aim of this work was to study the curing processes and properties of epoxy nanocomposites with CNTs obtained by float catalysis as a filler.

## 2. Materials and Methods

**Figure 1:**
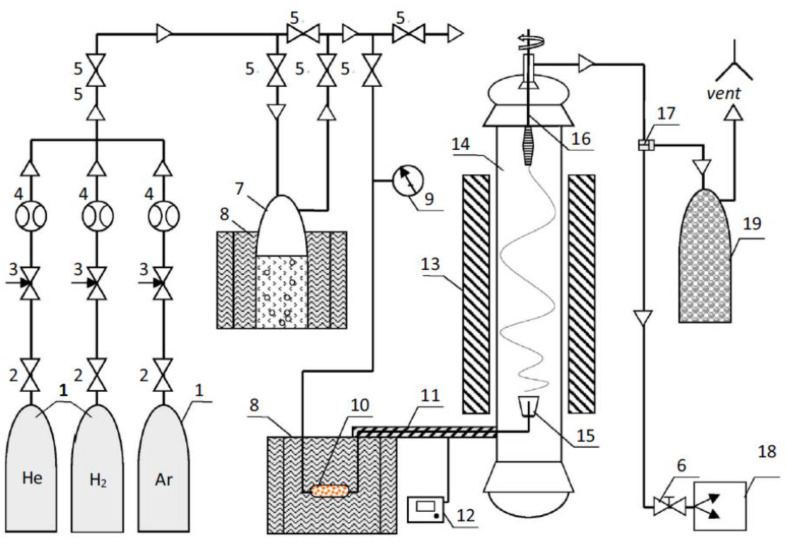
Schematic representation of a pilot plant for CNTs production as disclosed earlier in [9]: (1) gas cylinders; (2) control valves; (3) flow meters; (4) ball valves; (5, 6) check valves; (7) saturator with a mixture of ethanol/thiophene; (8) thermostat; (9) pressure gauge; (10) saturator with ferrocene; (11) pipeline of saturated raw materials; (12) control unit; (13) oven; (14) reactor; (15) inlet nozzle; (16) winding device; (17) valve; (18) vacuum pump; (19) dust filter. The Figure is adapted from [9], with permission from Elsevier (permission automatically granted in accordance with 2021 STM permission guidelines).

CNT felt was used as a nanofiller of epoxy nanocomposites and was obtained by the method of float catalysis [9] on the pilot plant for producing CNTs (Figure 1) at a temperature of 1150 °C in a stream of hydrogen from a mixture of ethanol, acetone and thiophene (activator) in mass ratio 1/1/0.03, respectively. Ferrocene was used as a precursor for the synthesis of CNT. 

ED 20 epoxy oligomer cured with 4,4′-diaminodiphenylsulfone (DADFS) was used as a binder for PCM. The PCM manufacturing process consisted of several stages:

- preliminary obtaining a dispersion of CNT felt in acetone,
- mixing the resulting dispersion with the epoxy composition ED 20 / DADFS,
- drying the samples from acetone,
- curing under the pressure according to the selected temperature program.

The dispersion of CNT felt in acetone was prepared by alternately using the Sonopuls-HD3400 submersible ultrasonic dispersant (exposure time 1 min) and IKAT 18 basic high-speed dispersant (3000 rpm, exposure time 1 min). Four cycles of exposure were used with breaks between cycles of 20 min for cooling during preliminary obtaining the dispersion of CNTs in acetone. The epoxy composition ED20/DDS was added to the resulting dispersion. Acetone was evaporated by holding the samples in an open container for 48 h at the room temperature and subsequent heat treatment in a vacuum box at a temperature of 60 °C for 2 h. The curing mode of the samples was chosen by the study of a gel formation using the method of dynamic mechanical analysis (DMA). The samples were pressed according to the regime: 140 °C for 1 h, 180 °C for 3 h. The thickness of the samples was set by the gaskets between the plates of the press.

Thus, there were four types of samples prepared. The features and the differences of the samples are presented in Table 1. It is important to note that the ratio of the components of the epoxy composition in ENC-1 (stoich) was stoichiometric. An excess amount of DDS was added during the manufacture of ENC-1 sample at the stage of mixing with the epoxy composition. The mass of the excess was equal to the mass of the initial CNTs. Also, CNT filler was additionally pre-dispersed in acetone in the presence of DDS curing agent during the preparation of ENC-2. The amount of DDS was equal to the mass of the CNTs.

MEASUREMENT PROCEDURES

The morphology and structural characteristics of the CNT felt were investigated by JEOL JSM-7600F scanning electron microscope (SEM) including thermal field emission with an accelerating voltage of 15 kV and resolution up to 1 nm, as well as by JEOL JEM-2010 transmission electron microscope (TEM). The content of residual catalyst in the felt, as well as the temperature of the onset of oxidation were determined by NETZSCH STA 449 F1 Jupiter (DTA/TGA) in the air flow of 70 mL/ min with a heating rate of 10 °C/ min from the room temperature to 800 °C. The specific surface area was determined by Autosorb-1c instrument and Ultrapyc 1200e gas pycnometer (Quantachrome Instruments). Temperature characteristics and changes in the enthalpy of various thermal processes of the initial epoxy composition and epoxy nanocomposites were obtained by differential scanning heat flow calorimeter (DSC) manufactured by MettlerToledo (Switzerland) in the air without the use of purge gas from the room temperature to 330 °C with a heating rate of 10 °C/ min. The temperature of the onset of the heat process on the DSC curve was determined as the point of intersection of the tangents to the peak and the baseline extrapolated to the inside of the peak. Changes in enthalpy (thermal effects) of various thermal processes were determined as the area above/ below the DSC curve. The glass transition temperature and the elastic modulus of the cured samples of the initial composition and epoxy nanocomposites were determined using a combination of dynamic mechanical analysis (DMA) and thermomechanical analysis (TMA) by thermomechanical analyzer by Mettler Toledo (Switzerland). The measurements were made in the air without using purge gas with a heating rate of 5 °C/ min from the room temperature to 240 °C. The measurement mode (type of deformation) was three-point bending. Free bending length was 10 mm. The dynamic load was from 0.05 N to 0.1 N with the frequency of 1 Hz. The glass transition temperature of the initial composition and epoxy nanocomposites were determined by TMA thermogram as the temperature of the onset of deformation caused by the transition of the polymer matrix from the glassy state to highly elastic state. The value of this temperature was determined as the intersection of the tangent to the initial linear part of the TMA curve with the tangent at the point of maximum strain rate.

The curing mode of epoxy nanocomposites was chosen on the basis of a study of the gelation process by DMA. The measurements were carried out on a thermomechanical analyzer by Mettler Toledo (Switzerland) in the air without the use of purge gas in isothermal conditions at 140 °C. The preheating rate from the room temperature to 140 °C was 3 °C/min. The measurement mode (type of deformation) was compression. A sample of the composition with CNTs was placed between two layers of quartz fabric, then between two layers of thin aluminum foil. The resulting construction was placed in a cup of dense aluminum foil between two quartz disks. The upper disk was located under the indenter dynamically loaded with a force of 0.1N to 0.3N with a frequency of 1 Hz. The gelation time was determined by the characteristic inflection on the experimental time dependence of the elastic modulus at DMA curve. The specific conductivity of the cured nanocomposites was determined by the van der Pauw method. Samples of epoxy nanocomposites were studied by TESCAN VEGA 3 XMU electron microscope in the secondary (SE) electron mode at magnifications from ×100 to ×40,000 according to MI 1.2.042-2011. The fracture of the samples was obtained after treatment with liquid nitrogen, then they were glued with conductive glue to the microscope holder. The surface of the samples was subjected to ion-plasma etching on a JFC-1100 (JEOL) installation followed by sputtering of gold ≤ 10 nm in a Q150R ES magnetron sputtering system (Quorum Technologies). 

## 3. Results

**Figure 2:**
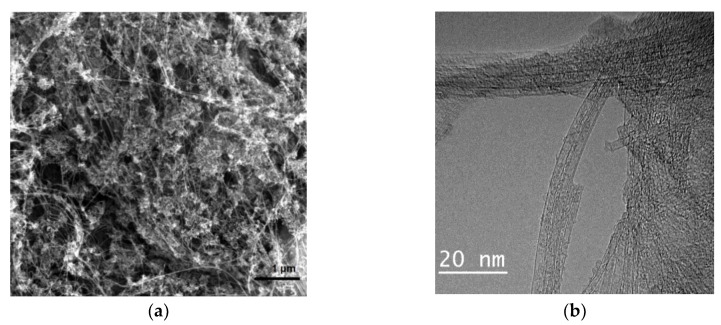
Electron microscopy images of the CNT used: (**a**) SEM; (**b**) TEM.

The study of CNT “felt” by electron microscopy showed that the structure of the main product is dominated by two-, three- and multi-walled nanotubes with a characteristic diameter of 2–40 nm. The tubes are mainly bundled (Figure 2).

**Figure 3:**
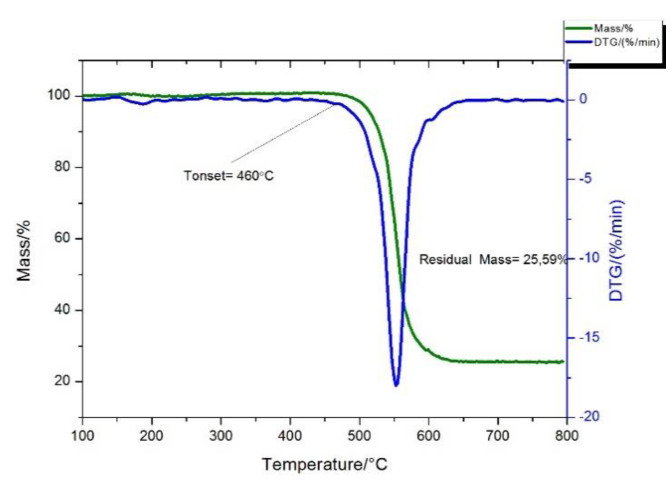
TG curve (green), DTG curve (green dotted), DTA curve (blue) of high-temperature oxidation of CNT felt.

The high-temperature oxidation of CNT felt occurs in one stage (Figure 3), the iron content in the obtained carbon product is about 18% by mass according to thermogravimetric analysis (TGA), the temperature of the onset of nanotube oxidation is 460 °C (Figure 3).

Sorption studies have shown that the specific surface area is about 200 m^2^/g.

**Figure 4:**
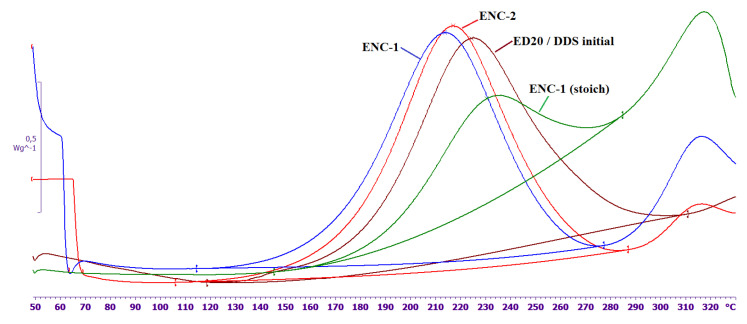
DSC curves of curing of ENC-1 (stoich), ENC-1, ENC-2 and the initial composition ED20/DDS. EXO direction is up.

The DSC curves of the curing, the temperature of the maximum heat, and the magnitude of the thermal effect for the epoxy nanocomposites and the original composition are shown in Figure 4 and Table 1. The ratio of ED 20/ DDS for the initial composition and ENC-1 (stoich) is stoichiometric. The mass of DDS excess was equal to the mass of the initial CNTs in ENC-1 and ENC-2. The mass ratio of felt and ED 20 in nanocomposites was 1:10. The magnitude of the thermal effect in epoxy nanocomposites was normalized to the mass of the epoxy oligomer.

It can be seen (Figure 4, Table 1) that curing reaction rate decreases when the stoichiometric composition (ENC-1 (stoich)) is used in the nanocomposite. The temperature of the max of thermal effect shifts to the region of higher temperatures by 5 °C and the specific thermal effect is almost halved.

An increase in the amount of curing agent in the system (ENC-1, ENC-2 samples) leads to an acceleration of the curing reaction. The temperature of the max of thermal effect shifts to low temperatures by 7–10 °C; the thermal effects of curing are compared for the initial composition and epoxy nanocomposites. At the temperatures far beyond the range recommended for the use of cured composition (i.e., beyond 230 °C) the destruction processes start showing themselves as peaks around 310 °C.

**Figure 5:**
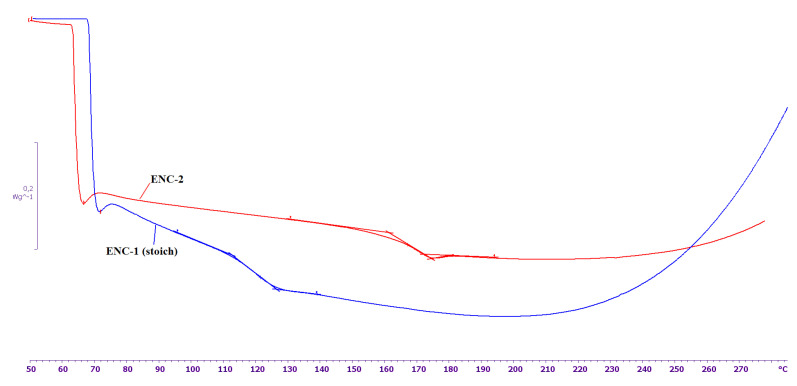
DSC curves of cured ENC-1 (stoich) and ENC-2.

The DSC curves of the cured samples ENC-1 (stoich) and ENC-2 are presented in Figure 5. As can be seen from this figure, the glass transition temperature of ENC-2 turns out to be 46 °C higher than for ENC-1 (stoich). It indicates the formation of a denser network of transverse chemical bonds in the system with an excess of curing agent compared to stoichiometric one. Therefore, it can be assumed that some of the functional groups of DDS are inactivated because of the interaction between DDS and the surface of CNT obtained by the FC-CVD method.

Moreover, according to thermogravimetric analysis in the air, the introduction of CNTs into the epoxy composition did not change the nature of the high-temperature oxidation of ENC-1 (stoich) in the region of the onset of mass loss compared to the initial epoxy composition. At the same time, a small increase in the temperature of the onset of oxidation was noted. It can be assumed that the filler plays a role of a protective agent that slows down the processes of high temperature oxidation. It leads to an increase in heat resistance compared to the initial polymer [10].

**Figure 6:**
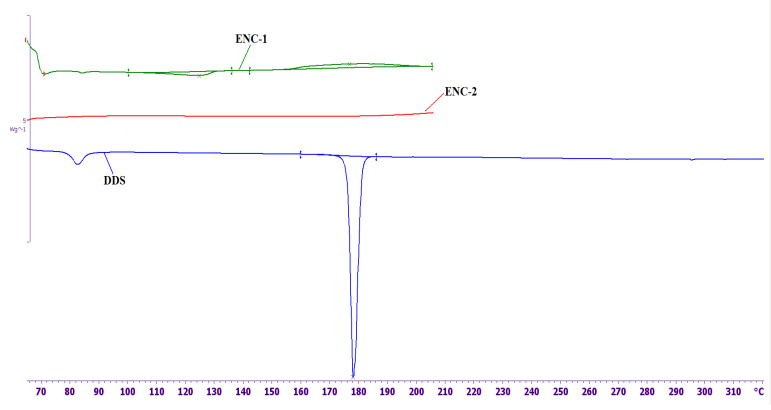
DSC curves of DDS and nanofillers (without epoxy oligomer) to obtain ENC-1 and ENC-2. The endothermic peak at 126 °C (the thermal effect is –10 J/g) can be associated with the melting of DDS localized on the surface of CNTs. Compared to pure DDS, the peak is shifted by 51 °C to the low-temperature region and is significantly broadened. The first exothermic peak is observed at a temperature of 177 °C, the thermal effect is 19.6 J/g; the second exothermic peak is observed at a temperature of 304 °C; the thermal effect is 320 J/g.

The DSC curves of nanofillers without epoxy oligomer that were used to prepare ENC-1 and ENC-2 and DSC melting curve of DDS are shown in Figure 6.

As can be seen from Figure 6, one endothermic peak and two exothermic peaks are observed on the DSC curve of ENC-1. The endothermic peak at 126 °C (the thermal effect is –10 J/g) can be associated with the melting of DDS localized on the surface of CNTs. Compared to pure DDS, the peak is shifted by 51 °C to the low-temperature region and is significantly broadened. The first exothermic peak is observed at a temperature of 177 °C, the thermal effect is 19.6 J/g; the second exothermic peak is observed at a temperature of 304 °C; the thermal effect is 320 J/g. It is necessary to note that for felt without a curing agent (not shown in the figure) there is only one exothermic peak at a temperature of 298 °C. This indicates a strong interaction of DDS with the surface of CNTs and partial DDS amorphization. The first exothermic peak is probably associated with the reaction of DDS with active centers on the surface of CNTs. The second exothermic peak, which is also observed on CNTs without DDS, is associated with the processes of thermooxidative destruction of active centers on the surface of CNTs.

**Figure 7:**
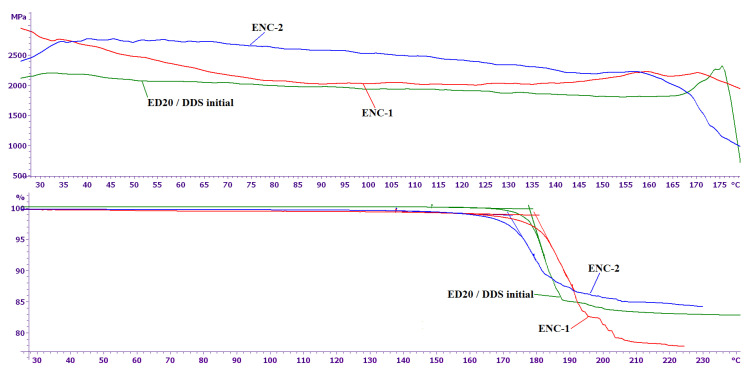
Temperature dependence of the elastic modulus (above) and relative deformation (below) for the unmodified ED20/ DDS system, ENC-1 and ENC-2.

After heat treatment of CNTs with DDS according to the regime mentioned before, neither the low-temperature endothermic peak, nor the first exothermic peak is observed, which suggests that DDS was immobilized at the surface of CNTS and thus became inactive in curing. As can be seen from the presented results, the elastic modulus of epoxy nanocomposites at the room temperature is 20–40% higher than the elastic modulus of the initial epoxy composition (Figure 7). The glass transition temperatures of the epoxy nanocomposites and the initial composition are practically equal and range from 170 to 180 °C (Figure 7).

**Figure 8:**
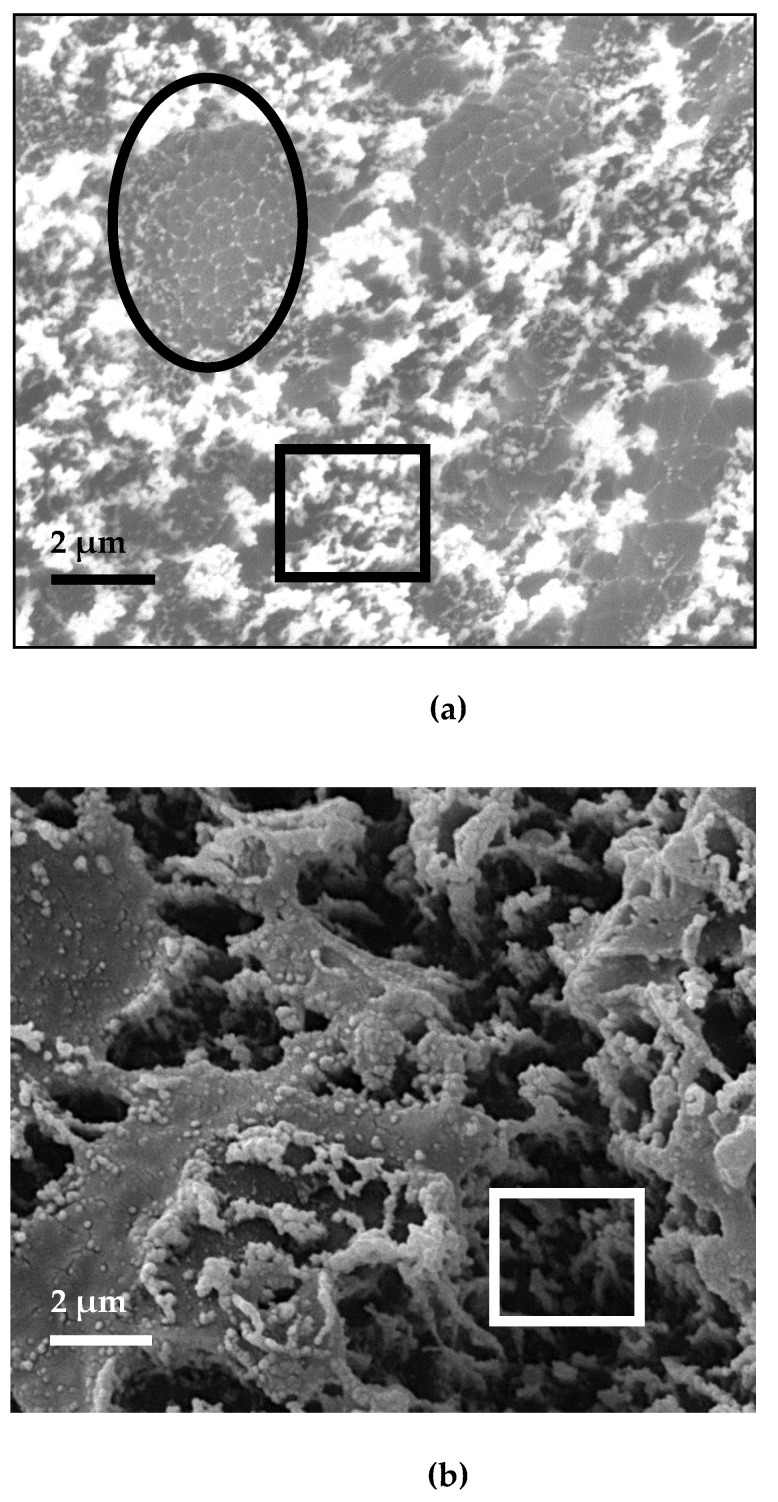
The SEM images: (**a**) ENC-1; (**b**) ENC-2.

One of the possible reasons for such a small change in the elastic modulus of the epoxy nanocomposite is a high degree of heterogeneity of their microstructure (Figure 8).

As can be seen from the above photographs, the ENC-1 contains fiber-like structures (Figure 8a, oval frame). Such structures probably include intergrowths of “long” CNTs obtained by the FC-CVD method. The microstructure of ENC-2 does not contain such fiber-like fragments (Figure 8b). CNTs of ENC-2 are coated with a layer of epoxy composition, which is typical for CNTs fabricated by the FC-CVD method after their functionalization in acid [11].

Both ENC-1 and ENC-2 have fragments that are typical for CNTs fabricated by CVD pyrolysis (square frame). Therefore, it can be suggested that the structure of CNT felt includes CNT fragments that are subject to destruction at lower stress than intergrowths of “long” CNTs.

Despite the high concentration of CNTs, the uncured ENC-2 can probably be used for the manufacture of electrically conductive glass and carbon plastics, as well as functional coatings. The specific conductivity of the cured ENC-2 is 4 S/cm.

## 4. Conclusions

Epoxy nanocomposites with float catalysis-produced CNT felt filler were prepared and studied. It was established that the presence of CNT felt decelerates the curing reaction and reduces its thermal effect compared to the initial epoxy composition in the case of a stoichiometric ratio of the components in epoxy nanocomposite. CNT felt accelerates the curing reaction in the case of an excess of curing agent, the thermal effect of such reaction is compared with the thermal effect of curing for the initial epoxy composition. It was shown that the curing agent can enter an exothermic reaction with the surface of CNTs. It leads to the individualization of CNTs in the epoxy nanocomposite and the formation of a polymer “coat” on them. It was found that the structure of the epoxy nanocomposites has a high degree of heterogeneity: the presence of fiber-like structures and individualized CNTs is observed together with the regions that are typical for epoxy nanocomposites with CNTs that are fabricated by CVD. Based on the studies performed, it is possible to predict the production of epoxy nanocomposites with outstanding mechanical and thermophysical properties. In particular, the uncured compositions already obtained in this work can be used for the manufacture of electrically conductive glass and carbon fiber reinforced plastics and functional coatings.

## Figures and Tables

**Table 1:** The curing characteristics of the starting composition and epoxy nanocomposites.

**No**	Sample Name	Description	Temperature of the Max of Thermal Effect (°C)	Specific Thermal Effect (J/g) *
1	ED 20/DDS initial	Initial composition ED 20/DDS, stoichiometric composition	224.5	307
				
2	ENC-1 (stoich)	Epoxy nanocomposite, stoichiometric composition ED 20/DDS	231	120
3	ENC-1	Epoxy nanocomposite, excess of DDS at the stage of mixing the components	214	293
4	ENC-2	Epoxy nanocomposite, excess of DDS at the stage of dispersion in acetone	217	317

* the heat released per 1 g of composition.

## Data Availability

The data presented in this study are available on request from the corresponding author. The data are not publicly available due to institutional restrictions.
